# Acute compartment syndrome of the lower limb following childbirth: a case report

**DOI:** 10.1186/s13256-020-02459-w

**Published:** 2020-09-04

**Authors:** Sharon Coulton, Sally Bourne, Simon Catliffe, Roderick Brooks, David Jollow

**Affiliations:** 1 School of Nursing and Midwifery, Western Sydney University, Sydney, Australia; 2 Hornsby Ku-Ring-Gai Hospital, Hornsby, NSW 2077 Australia; 3 Obstetrics and Gynaecology, Hornsby Ku-Ring-Gai Hospital, Hornsby, NSW 2077 Australia

**Keywords:** Case report, Acute compartment syndrome, Childbirth, Cesarean, Obstetrics, Postpartum pain

## Abstract

**Background:**

Acute compartment syndrome is a limb-threatening and occasionally life-threatening emergency that is rarely reported as a complication following childbirth. Prompt diagnosis is crucial to avoid permanent functional restriction or even the loss of the affected limb. Clinical signs and symptoms might be nonspecific, especially in the early stages; therefore, knowledge of predisposing risk factors and signs and symptoms of acute compartment syndrome is necessary to prevent long-term complications and amputation.

**Case presentation:**

This paper presents a case of a 26-year-old primiparous Sri Lankan woman who developed acute compartment syndrome of the lower right limb following childbirth by cesarean section.

**Conclusion:**

Acute compartment syndrome is an important differential diagnosis in the setting of sudden onset of lower limb pain following childbirth. Predisposing factors for its manifestation within an obstetric environment are augmented labor, the lithotomy position, postpartum hemorrhage, hypotension following epidural analgesia, and the use of vasoconstrictive agents. If left undiagnosed and untreated, acute compartment syndrome may cause permanent neurovascular deficit, leading to a poor functional result, tissue ischemia, limb amputation, and rhabdomyolysis. If severe, and in large compartments, it can lead to renal failure and death. Alertness and a high index of clinical suspicion for the possibility of acute compartment syndrome are required to avoid a delay in diagnosis, and intracompartmental pressure measurement can be used to confirm the diagnosis.

## Statement of significance

### Problem

Acute compartment syndrome (ACS) is not currently recognized by midwives and obstetricians as a potential complication that may occur following childbirth. The published literature estimates a prevalence of 2 in 10,000 births [1]; however, its true incidence is unknown.

### What is already known

Alertness and a high index of clinical suspicion are required to avoid delayed diagnosis and permanent neurovascular deficit.

### What this report adds

This case report highlights the need for midwives and obstetricians to have an understanding of the signs and symptoms of ACS to prevent delayed diagnosis and its consequences for the postpartum woman.

## Background

ACS of the limb is a limb-threatening and occasionally life-threatening emergency. It is caused by an increased intracompartmental pressure (ICP) that causes a decrease in perfusion pressure, leading to hypoxemia of the tissues [2]. A decrease in tissue perfusion can lead to irreversible necrosis, which may result in functional impairment, loss of limb, and, in rare cases, death [2]. ACS most frequently occurs after a traumatic event; however, up to 30% of cases develop without evidence of fracture [2]. Other factors that have been associated with ACS include ischemia-reperfusion injury; hemorrhage; phlegmasia cerulea dolens; vascular puncture in patients with bleeding disorders; intravenous/arterial drug injection; and soft tissue injury resulting from prolonged limb compression due to lithotomy positioning during surgery, constricting casts or wraps, crush injury, or burns [2, 3]. ACS has also been associated with nephrotic syndrome, rhabdomyolysis, bleeding disorders, iatrogenic factors, and infections such as *Streptococcus* spp. [2].

The symptoms of ACS include swelling, pain on passive stretch, pain out of proportion to the injury, paresthesia, and paresis or paralysis [4]; however, it is important to note that not all characteristic of ACS may be present [5]. Clinical signs such as pallor, reduced capillary return, and absent peripheral pulses are late signs of ACS, and therefore the presence of distal pulses should not be relied on to exclude a diagnosis of ACS [4]. Significantly increased creatine kinase (CK) levels in the blood are also not a good indicator for early diagnosis of ACS, because they may indicate severe muscle damage or ischemia [2–4, 6, 7].

Because ACS is rarely reported as a complication in the obstetric literature, it is not currently recognized by midwives and obstetricians as a potential complication that may occur following childbirth. Available case reports indicate that it may occur at any stage during labor, birth, or the immediate postpartum period. The published literature estimates a prevalence of 2 in 10,000 births [1]; however, its true incidence is unknown. It has been reported in the settings of both vaginal [8–12] and cesarean births [13–19] and has been diagnosed on the basis of clinical evaluation and occasionally ICP measurement.

Possible risk factors for ACS of the limb in an obstetric context has been associated with, but is not limited to, postpartum hemorrhage (PPH) [13, 16, 18, 19], epidural analgesia [16, 17], hypotension [9, 12, 17], vasoconstrictive agents [8, 13, 16–18], the lithotomy position [8], postpartum eclampsia [11, 16], and a combination of these factors [8, 9, 13, 16, 17].

Augmentation and induction of labor with oxytocics, PPH, use of vasoconstrictive agents, lithotomy position, and epidural analgesia and related hypotension occur regularly in an obstetric environment, and a combination of these factors may predispose a woman to develop postpartum ACS of the limb. Because the functional outcome after ACS is directly related to urgent surgical intervention, it is essential for midwives and obstetricians to be aware of the potential for an ACS to develop and to consider it as a differential diagnosis when symptoms are present.

This report presents a case study of delayed diagnosis of ACS of the right lower leg following childbirth by cesarean section. The purpose of this report is to prevent further cases of delayed diagnosis by raising awareness of the need to have a high index of suspicion when signs and symptoms of ACS are present. Reporting of all cases of ACS following childbirth, regardless of the outcome, is strongly encouraged so that knowledge and awareness of the possibility of this complication are increased among midwives and obstetricians who provide care for women during labor, birth, and the immediate postpartum period. To prevent cases of delayed diagnosis, it is necessary for ACS to be recognized as a differential diagnosis and a potential complication in obstetrics when possible risk factors are present.

## Case presentation

A 26-year-old Sri Lankan woman in early labor presented to the birthing unit at 40 weeks and 6 days of gestation with spontaneous onset of labor and rupture of membranes. She had experienced threatened premature labor at 22 weeks and 6 days of gestation, but the pregnancy was otherwise uncomplicated.

The woman progressed well into established labor; however, labor slowed following administration of morphine. Augmentation of labor was commenced with 10 IU of oxytocin (Syntocinon; Mylan Health Pty Ltd, Millers Point, Australia) in 1 L of Hartmann’s solution, after which labor progressed well. Seventeen hours following admission to the hospital, the laboring woman had not progressed beyond 7cm dilation. Epidural analgesia was commenced, but due to “failure to progress” in the first stage of labor and a nonreassuring fetal heart rate, the woman gave birth by cesarean section. A healthy male infant was born following 16 hours and 51 minutes of established labor, and an estimated blood loss of 450 ml was recorded.

Twelve hours following the cesarean section, the woman started to experience pain in her lower right leg, and by 28 hours, she had developed clinical symptoms of an ACS. She was treated with antibiotics for possible cellulitis. Her symptoms did not improve, and a Doppler ultrasound examination was performed to exclude deep venous thrombosis (DVT) but showed no remarkable findings. ACS was definitively diagnosed on postpartum day 10 and confirmed by magnetic resonance imaging. The ACS was managed nonsurgically, and the woman remained in the hospital with her baby for 15 days following the birth. A detailed timeline of events and clinical management following the cesarean section is provided in Table 1.

**Table 1:** Timeline of events following birth

Time	Clinical details
1.5 hours	Returned to the postnatal ward following an emergency cesarean section; full sensation and movement were noted in both legs.
12 hours	Onset of pain and swelling in right lower leg.
14 hours	The postpartum woman complained of pain in her right shin. An obstetrics and gynecology assessment revealed extreme pain on mobilization and weight bearing in the lower right leg, with worsening pain upon movement over the tibialis anterior with plantar flexion. A physical examination revealed a decreased range of motion in the right foot and decreased extension of the right great toe. Minimal pitting edema was noted on the right foot, and capillary refill was normal in both feet. A small and very painful soft lump was palpable over the anterior midshin area of the right leg. Range of motion in both knees and ankles was normal, as were the lower limb reflexes and pedal pulses. No calf tenderness or skin changes were noted, and skin temperature was normal. Sensation was normal, and the postpartum woman was afebrile. The impression at the time was deemed to be muscle-related pain, and therefore she was commenced on analgesia and encouraged to mobilize and to elevate the leg on a pillow. A deep venous thrombosis was not considered at this stage, because there was no calf tenderness. A heat pack was applied to the lower right leg and antiembolism stockings were applied to both legs.
23 hours	Swelling in the right leg and foot had worsened, and the postpartum woman experienced a burning sensation along the muscle. By this stage, she was no longer able to bear weight.
28 hours	The antiembolism stockings were removed due to increased pain, and this resulted in sudden and extreme pain and further swelling in the right lower leg and foot. Assessment revealed that the right leg and foot were very swollen and the foot was curving inward and was painful to touch. The postpartum woman was unable to move her toes or lift her foot due to pain, and a review was conducted by the obstetrics and gynecology registrar. By this stage, the woman was in extreme pain and reported constant throbbing in the leg. Examination revealed a decreased range of motion in the right foot; the right foot was markedly more swollen than the left; and the anterior right leg and anterior foot were very tender to touch. Pedal pulses were palpable, and perfusion to the toes was noted. The nodule over the anterior midshin remained palpable, and the skin now appeared red. There was no reported calf pain, and she remained afebrile. Cellulitis was diagnosed, and a blood sample collected at this time revealed deranged liver function test results. The woman was then commenced on intravenous antibiotics and enoxaparin sodium (Clexane; Sanofi-Aventis, Macquarie Park, Australia), an anticoagulant, prophylactically because she was now immobile. Subcutaneous morphine was administered with no effect, and the antiembolism stocking was unable to be reapplied to the right leg due to extreme pain. Six hours following this acute stage, the postpartum woman reported feeling more comfortable, and her right leg was less swollen and less tender; however, the limb remained red. An anesthetic review at this time excluded an epidural-related cause for the ongoing right great toe weakness.
Day 2	There was still visible swelling and worsening of pain after mobilizing; therefore, the patient remained on bedrest. Clexane was changed to a therapeutic dose, and analgesia was continued (Endone [oxycodone hydrochloride], Alphapharm Pty Ltd, Millers Park, Australia; Panadol [acetaminophen], GlaxoSmithKline Australia, Ermington, Australia; and Voltaren [diclofenac], GlaxoSmithKline Australia). Venous Doppler ultrasound was performed to exclude deep venous thrombosis, and the findings were unremarkable. The leg was more inflamed and reddened following the Doppler ultrasound, and the postpartum woman was now completely unable to bear weight on the right leg.
Day 3	Some improvement in pain and redness was noted, but weakness of the right great toe remained. The postpartum woman was still unable to hyperflex the right great toe, and the results of her liver function tests were more deranged. Clexane was reverted to a prophylactic dose, and antibiotics were changed again; the woman remained afebrile. A second anesthetic examination confirmed ongoing weakness of the great toe, and sensation was intact. The differential diagnosis at this stage included possible neurological problems, cellulitis, or gout.
Day 4	Skin redness had started to improve, but mobility was limited due to pain. Neurological symptoms remained unchanged, and some reduction in plantar adduction was noted. Upon examination by a neurologist, the leg was tender and swollen below the knee. There was severe pain upon examination, and the neurologist was unable to check right inversion due to the pain. Sensation was found to be decreased to the peroneal region. The differential diagnoses queried by the neurologist included right peroneal nerve palsy, pressure effect, compartment syndrome, and cellulitis. The postpartum woman was referred to the infectious diseases team for management of cellulitis and for a vascular opinion regarding possible compartment syndrome. Antibiotics were changed again, and a surgical review deemed that symptoms were not diagnostic of compartment syndrome.
Day 4 (95 hours)	By this stage, the postpartum woman reported numbness between the right great toe and the second toe, a new symptom.
Day 5	An examination revealed increased redness and loss of sensation over the first web space of the right foot. The postpartum woman continued to complain of increased pain. The diagnosis at the time was worsening cellulitis and worsening of neurological symptoms secondary to the worsening cellulitis. Antibiotics were changed again, and the woman was continued on Clexane.
Day 7	Symptoms of cellulitis remained unchanged. Antibiotics were changed again in consultation with the infectious diseases team.
Day 8	Neurological symptoms remained unresolved, so a neurologist was consulted again.
Day 10	Magnetic resonance imaging was performed, and the findings were consistent with acute compartment syndrome.
Day 11	Orthopedic consultation was obtained. There were clinical features of a compartment syndrome affecting the anterior compartment of the right lower leg. An examination showed foot drop and paresthesia along the deep and superficial peroneal nerves. Pain had moderated by that time, but there was still evident swelling of the anterior compartment with associated tenderness and some dusky erythema. There was no active contraction of the great toe extensor, and there was reduced extensor function of the lesser toes and the tibialis anterior. The anterior compartment pressure was measured as 19 mmHg, and pressure in the peroneal compartment was 23 mmHg. Following consultation with other orthopedic and vascular surgeons at a tertiary referral hospital, the compartment syndrome was managed nonsurgically, given that 11 days had elapsed since the onset of symptoms and a fasciotomy at this stage would not prevent any further damage.
Days 11–15	The woman was commenced on physiotherapy and fitted with a foot drop splint and a rollator frame to assist with mobilization. Intravenous antibiotics were continued until discharge from the postnatal ward.
Day 15	The woman was discharged to home on a course of oral antibiotics.

### Orthopedic report

Over the following months, the patient experienced improvement in tibialis anterior function. The main residual deficit was contracture of the extensor hallucis longus and the extensor digitorum longus to the lateral two toes. The patient had altered sensation over the dorsum of the first web space. Nerve conduction studies were conducted six weeks following the ACS and indicated a lesion of the superficial and deep branches of the right peroneal nerve, more markedly affecting the deep branch.

Fifteen months following the original diagnosis, the woman underwent tendon surgery. Percutaneous tenotomies were performed of the extensor tendons to the fourth and fifth toes, and a Z-lengthening of the extensor hallucis longus tendon was performed to correct the dorsiflexion contracture that had developed following the ACS. The woman recovered well from this procedure.

At long-term follow-up (5 years), the woman walks normally. She has no active dorsiflexion of the great toe. She can walk in bare feet but must be careful not to trip over the hallux, so she prefers closed-in footwear. Pain in the affected leg has diminished but not completely resolved. Dorsiflexion of the ankle is slightly reduced in power and in range, and there is slight persistent anterolateral wasting of the leg. There is slight weakness of extension of the lesser toes, and a contracture is evident when the ankle is plantarflexed.

### Physiotherapy report

Five years following a delayed diagnosis of ACS, there are some ongoing deficits in the right leg secondary to the ACS, including reduced ankle dorsiflexion power and a complete loss of function of the extensor hallucis longus following from release of the distal tendon due to contracture development. These ongoing deficits, along with compensatory movement patterns, have contributed to the development of further issues higher up in the kinetic chain. Findings of an assessment at this stage included reduced lumbar spine active range of motion (especially extension and left lateral flexion) with sharp right-sided lumbopelvic pain. There are signs of left sacral torsion and reduced right lower limb proprioception and lumbopelvic stability (poor single-leg balance and ability to perform single-leg bridge). Ongoing physiotherapy treatment now focuses on addressing these issues in the pelvis and spine.

## Discussion

ACS of the limb is a relatively rare complication in obstetrics and has an estimated prevalence of 2 in 10,000 births [1]. Several predisposing risk factors have been described in the published case reports, such as PPH [13, 16, 18, 19], epidural analgesia [16, 17], hypotension [9, 12, 17], vasoconstrictive agents [8, 13, 16–18], lithotomy position [8], and postpartum eclampsia [11, 15]; however, a combination of factors may also be responsible [8, 9, 13, 16, 17].

PPH has been identified as a contributing factor for the development of ACS of the limb in four obstetric case reports [13, 16, 18, 19] and was also described in a further three case reports [9, 10, 12]; however, PPH did not occur in one case [17]. In our patient’s case, the estimated blood loss was recorded as 450 ml. Although this does not meet the criteria for PPH (500 ml), it is important to note that our patient’s hemoglobin levels decreased from 128 g/L at the time of augmentation of labor to 88 g/L on day one following the birth. The woman in this case study was of small stature, and it is possible that her estimated blood loss was sufficient to cause hemodynamic compromise [20], although it is also possible that her estimated blood loss at the time of the cesarean section may have been underestimated [17].

Administration of vasoactive drugs is a known predisposing factor for ACS and has been reported in most of the published obstetric cases [8, 9, 11, 13, 16–18]. In addition to venous stasis of lower extremities, a physiologic event in all pregnancies, vasoactive agents may contribute to the deterioration of circulation [8]. In the present case study, Syntocinon, a uterotonic, was used for augmentation of labor and also immediately after birth to prevent PPH.

Long-lasting hypotension and intraoperative hypotension induced by anesthetic agents have been reported as factors contributing to ACS in an obstetric context [8, 9]. Hypotension is described in three of the reported cases [9, 12, 18], and another author has suggested the possibility of an unnoticed hypotensive episode during a cesarean delivery [17]. The woman in the present case report was continuously monitored and developed intraoperative hypotension shortly following epidural insertion, and metaraminol, which also causes vasoconstriction, was administered in the operating theater.

The lithotomy position is well established in the literature as a predisposing factor for ACS of the limb [21–24]. This position increases the ICP and elevation above heart level, in addition to the head-down tilt, reduces limb perfusion, leading to ischemia [9]. Compartment syndrome usually develops from the reperfusion injury that occurs after the lithotomy position has been ceased [8, 9, 13]. Nonobstetric literature suggests that the incidence of ACS may be as high as 1 in 3500 surgical patients who are placed in the lithotomy position [25]. Obstetric cases that describe the use of lithotomy position are rarely reported in the literature, however, and only two of the reported cases identified the use of the lithotomy position [8, 9]. It is important to note that the woman in the present case study was not placed in the lithotomy position at any time during labor, supporting the possibility that a combination of factors in an obstetric context, rather than individual factors alone, may have contributed to the development of postpartum ACS of the limb.

The use of pneumatic calf compressors has been implicated in the development of ACS in the gynecologic and urologic literature [21, 26]; however, only one obstetric case identifies its use [13]. In the present case study, pneumatic calf compressors, as well as antiembolism stockings, were used for DVT prophylaxis following the cesarean section. Ill-fitting antiembolism stockings have been identified in the literature as a cause of lateral leg compartment syndrome [27], but this has yet to be described in other obstetric case reports.

Anticoagulation treatment following surgery, such as for DVT prophylaxis, may also contribute to the development of ACS [6, 23]. The use of enoxaparin sodium (Clexane; Sanofi-Aventis, Macquarie Park, Australia) has been described in the nonobstetric literature to spontaneously cause serious bleeding associated with ACS [28]. Only one other obstetric case report described the use of heparin [10]. The woman in the present case report was commenced on Clexane after the onset of acute pain, and it is possible that this may have exacerbated the already-developing ACS.

Although not previously identified as a predisposing factor for ACS in obstetrics, it is possible that the application of heat to a limb may contribute to or exacerbate a developing ACS [8]. In the present case study, a heat pack was applied to the right leg after the initial examination when muscle-related pain was considered. Authors of the only other obstetric case report describing the use of a heat pack suggested that the superficially applied heat may have changed the circulation of the extremity, further diminishing intracompartmental perfusion [8].

Rhabdomyolysis, a serious condition caused by muscle injury, may occur if the diagnosis of ACS is not made in time for urgent fasciotomy. The presence of high CK levels in the blood may indicate severe muscle damage or ischemia and may cause acute renal failure [3, 5, 9]. In the absence of clinical signs, raised CK levels could indicate an unsuspected ACS [6]; however, because rhabdomyolysis can lead to acute renal failure, and can be life-threatening [9], elevated CK levels should not be relied on for an early diagnosis of ACS [2–4, 6, 7], and creatinine clearance and plasma creatinine are late-stage markers of kidney damage that has already occurred [29]. In the present case study, the CK level on postpartum day 10 was 1150 U/L (normal range 30–190 U/L). CK readings commenced on day 10 only, and therefore it is impossible to determine the peak levels.

## Conclusion

In an obstetric setting, the following clinical features should give rise to suspicion of the diagnosis of ACS: severe pain in the affected compartment; persistent sensory loss in the foot after the epidural has worn off; persistent weakness of the foot or ankle, such as foot drop or toe drop; and swelling, tenderness, or erythema of the affected leg compartment. Should ACS be suspected, orthopedic or surgical consultation should be requested urgently. Compartment pressures can be measured if the clinical diagnosis is in doubt, and an early fasciotomy can prevent permanent nerve and muscle damage. This case highlights the need for maternity care providers to be aware of acute compartment syndrome as a potential obstetric complication following childbirth. Midwives and obstetricians should have knowledge of the predisposing factors for ACS, and its signs and symptoms, so that a delayed diagnosis and its subsequent consequences can be prevented.

## Abbreviations

ACS: Acute compartment syndrome
CK: Creatine kinase
DVT: Deep venous thrombosis
ICP: Intracompartmental pressure
PPH: Postpartum hemorrhage
